# Maternal Complications Associated with Primiparous Adolescent Pregnancies

**DOI:** 10.3390/jcm15124663

**Published:** 2026-06-16

**Authors:** Mihai Gabriel Marin, Ioana Păvăleanu, Ana-Maria Haliciu, Andreea Ioana Pruteanu, Raluca Mihaela Gemanariu, Cornelius Eduard Carp, Sorana Caterina Anton, Raul Andrei Crețu, Emil Anton

**Affiliations:** 1 “Grigore T. Popa” University of Medicine and Pharmacy, 16 University Street, 700115 Iasi, Romania; anna_cefalan@yahoo.com (A.-M.H.); andreea.ioana.dragu@gmail.com (A.I.P.); raluca_gemanariu@yahoo.com (R.M.G.); westfallia@gmail.com (C.E.C.); sorana.anton@yahoo.com (S.C.A.); c.raulandy@yahoo.ro (R.A.C.); emil.anton@yahoo.com (E.A.); 2 “Elena Doamna” Clinical Hospital of Obstetrics and Gynecology, 49 Elena Doamna Street, 700398 Iasi, Romania; 3 Department of Mother and Child Medicine, “Grigore T. Popa” University of Medicine and Pharmacy, 16 University Street, 700115 Iasi, Romania; 4 Department of Gynecology, “Grigore T. Popa” University of Medicine and Pharmacy, 16 University Street, 700115 Iasi, Romania; 5 Department of Morpho-Functional Sciences I, “Grigore T. Popa” University of Medicine and Pharmacy, 16 University Street, 700115 Iasi, Romania; 6 Neurology Department, “Grigore T. Popa” University of Medicine and Pharmacy, 16 University Street, 700115 Iasi, Romania

**Keywords:** adolescent pregnancy, prenatal care, maternal complications, obstetrical complications, pregnancy outcomes, perinatal risk

## Abstract

**Background**: Adolescent pregnancy is associated with increased maternal and neonatal morbidity, particularly in the context of inadequate prenatal care. Understanding the distribution of maternal and obstetrical complications in this population is important for improving clinical management and pregnancy outcomes. **Methods**: This retrospective observational study included primiparous adolescent patients (≤18 years) and focused on the assessment of maternal and obstetrical complications. The analysis compared the frequency of these complications between adolescents with adequate prenatal care and those without adequate prenatal follow-up, aiming to identify the most common complications and their distribution according to antenatal care status. **Results**: Significant differences were identified between groups. Maternal infections were more frequent among patients without adequate prenatal care (24.1% vs. 9.3%, *p* = 0.039). Hemorrhage was significantly more frequent among patients with adequate prenatal care (59.3% vs. 35.2%, *p* = 0.012). Among obstetrical complications, cephalopelvic disproportion showed a significant association with prenatal care status (*p* = 0.034), occurring more frequently in patients without antenatal follow-up. Hypertensive disorders, including gestational hypertension and preeclampsia, were identified only among patients without adequate prenatal care; however, no statistically significant association was observed (*p* = 0.118). Placenta previa (*p* = 0.057) and placental abruption (*p* = 0.495) were also observed only among patients without adequate prenatal care. **Conclusions**: Primiparous adolescent patients without adequate prenatal care showed a higher frequency of maternal and obstetrical complications, particularly infections and delivery-related disorders. Prenatal monitoring was associated with earlier identification and management of maternal and obstetrical risk factors. These findings highlight the importance of improving access to antenatal care among adolescent populations.

## 1. Introduction

Adolescent pregnancy remains a major public health concern worldwide due to its association with increased maternal, obstetrical, and neonatal risks. According to global health data, pregnancies occurring at a young maternal age are more frequently complicated by adverse outcomes such as preterm birth, low birth weight, hypertensive disorders, increased rates of operative delivery, maternal infections, and obstetrical complications requiring urgent intervention [1,2,3]. These risks are influenced not only by biological factors, including incomplete physical and reproductive maturation, but also by important social determinants such as limited access to healthcare, low educational level, and socioeconomic vulnerability [4,5].

Prenatal care represents a cornerstone of modern obstetrics and plays a fundamental role in the early identification, prevention, and management of pregnancy-related complications [6]. Adequate antenatal monitoring allows timely diagnosis of conditions such as infections, hypertensive disorders, fetal growth abnormalities, and placental complications, which may significantly influence both maternal and neonatal outcomes. Regular prenatal follow-up also facilitates appropriate counseling and delivery planning, potentially reducing the need for emergency interventions and improving pregnancy outcomes [7,8,9].

Despite its well-established benefits, access to prenatal care remains uneven, particularly among adolescent populations. Younger pregnant patients are more likely to initiate prenatal care late or to have inadequate antenatal follow-up. This may be related to multiple factors, including limited health literacy, social stigma, fear of disclosure, reduced autonomy in seeking healthcare, and barriers related to healthcare accessibility. Differences in socioeconomic status and healthcare infrastructure may further influence prenatal care utilization [3,10,11].

The relationship between prenatal care and pregnancy outcomes remains complex, as some complications are multifactorial and may occur even in well-monitored pregnancies. The impact of antenatal care may therefore vary depending on the type of complication, with a greater influence observed in conditions that benefit from early detection and timely management [12,13].

In adolescent pregnancies, this relationship may be particularly important because younger patients frequently present with both biological vulnerability and social risk factors. At the same time, the extent to which prenatal care may influence the distribution of maternal, obstetrical, and neonatal complications in adolescent populations remains insufficiently explored. Understanding these associations may contribute to improving both clinical management and public health strategies aimed at reducing maternal and neonatal morbidity [14].

The aim of the present study was to evaluate maternal, obstetrical, and neonatal outcomes among primiparous adolescent patients according to prenatal care status. By comparing patients with adequate prenatal care and patients without adequate prenatal care, the study aimed to identify differences in the distribution of pregnancy-related complications and to evaluate patterns of risk associated with inadequate antenatal follow-up.

## 2. Materials and Methods

**Figure 1:**
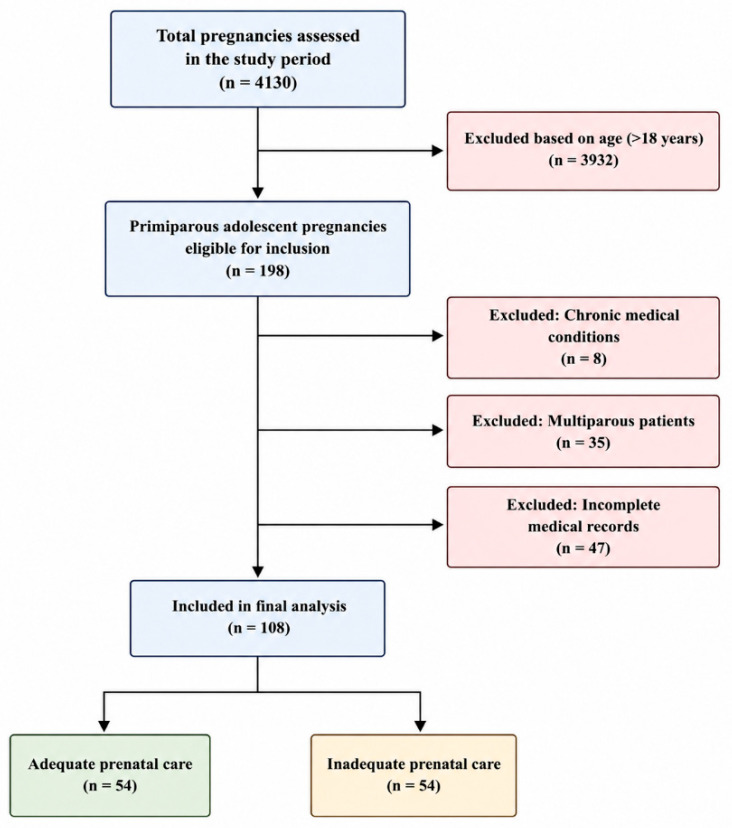
Flowchart of patient selection and allocation according to prenatal care status.

The study was conducted as a multicenter retrospective observational study over a two-year period, between January 2023 and December 2024. Cases were collected consecutively from two tertiary obstetrics and gynecology centers located in the Moldavia region of Romania (Figure 1). Eligible adolescent pregnancies meeting the inclusion criteria during the study period were identified through hospital medical records and included in the analysis.

The primary aim of the study was to evaluate maternal complications occurring in primiparous adolescent pregnancies and to assess potential differences between patients who received adequate prenatal care and those who did not. The study aimed to explore obstetrical complications and pregnancy and neonatal outcomes in relation to prenatal care status, in order to better characterize risk patterns within this vulnerable population.

### 2.1. Study Population

The study included adolescent pregnant patients who met the following inclusion criteria:

- Maternal age ≤ 18 years at the time of delivery;
- Primiparous status;
- Availability of complete medical records.

Patients were excluded if they met any of the following criteria:

- Maternal age > 18 years;
- Incomplete or missing clinical data;
- Presence of pre-existing maternal chronic conditions (such as chronic hypertension, pregestational diabetes, or significant renal or cardiovascular diseases);
- Multiple pregnancies or known fetal malformations.

### 2.2. Data Collection

Data were retrospectively extracted from the medical records of patients managed during the study period. The collected information included demographic and socioeconomic characteristics such as maternal age, area of residence, smoking status, obesity, maternal educational level, and family educational background.

Data regarding prenatal care status were also collected, distinguishing between patients with adequate prenatal care and those with inadequate or absent prenatal care. Classification was based exclusively on information documented in the medical records. Patients were considered to have received adequate prenatal care when regular antenatal follow-up throughout pregnancy was documented, including completion of the major recommended prenatal evaluations and investigations. These included first-trimester ultrasound confirmation of pregnancy, first-, second-, and third-trimester fetal morphology assessments, second-trimester ultrasound evaluation performed at approximately 17 weeks of gestation, first-trimester combined screening (double test) or second-trimester biochemical screening (triple test), OGTT performed between 24 and 28 weeks of gestation, routine laboratory investigations throughout pregnancy, regular obstetrical follow-up after 36 weeks of gestation, and cardiotocographic monitoring during the final weeks of pregnancy until delivery. Patients who did not complete the majority of these recommended assessments, had irregular antenatal surveillance, or presented for delivery without documented prenatal monitoring were classified as having inadequate or absent prenatal care.

Clinical data included maternal complications such as gestational hypertension, preeclampsia, anemia, infections, laboratory parameters including hemoglobin and hematocrit values, and OGTT results. Obstetrical complications included placenta previa, placental abruption, labor dystocia, cephalopelvic disproportion, and hemorrhage. Gestational hypertension and preeclampsia were analyzed as mutually exclusive categories.

Maternal infections were analyzed as a composite variable and included the main clinically documented infectious complications identified during pregnancy, particularly urinary tract infections, vaginal infections, and chorioamnionitis, as recorded in the medical records. Due to the retrospective design of the study, infections were classified according to the documented obstetrical diagnosis rather than microbiological confirmation in all cases. Hemorrhage was defined as clinically documented obstetrical bleeding occurring during delivery or in the immediate postpartum period. Hemorrhagic events were further categorized according to the documented obstetrical cause. Among patients with hemorrhage, 15 cases were associated with cesarean delivery, 10 cases were related to cervical lacerations, and the remaining 26 cases occurred following placental expulsion, mainly in association with difficult placental delivery, retained placental tissue, or uterine atony. Hemoglobin values used for anemia classification were obtained from routine laboratory investigations performed prior to delivery. Pregnancy and neonatal outcomes were assessed using variables such as gestational age at delivery, intrauterine growth restriction, birth weight, fetal distress, neonatal respiratory distress, Apgar score at 5 min, and neonatal jaundice. Gestational age at delivery was categorized as term (37–42 weeks of gestation) or preterm (<37 weeks) (Table 1).

### 2.3. Management During Follow-Up

Maternal complications were categorized into medical and obstetrical conditions. Medical complications included gestational hypertension, preeclampsia, anemia, and maternal infections, while obstetrical complications comprised placenta previa, placental abruption, labor dystocia, cephalopelvic disproportion, and hemorrhage. Selected fetal and neonatal outcomes were additionally recorded in order to evaluate the potential association between maternal and obstetrical complications and pregnancy evolution. These variables included preterm birth, intrauterine growth restriction, fetal distress, neonatal respiratory distress, Apgar score at 5 min, birth weight, and neonatal jaundice (Table 2).

### 2.4. Clinical Criteria

Clinical definitions were established according to internationally accepted obstetrical guidelines. Gestational hypertension was defined as systolic blood pressure ≥ 140 mmHg and/or diastolic blood pressure ≥ 90 mmHg occurring after 20 weeks of gestation in the absence of proteinuria. Preeclampsia was defined as gestational hypertension associated with proteinuria (≥300 mg/24 h) and/or signs of maternal organ dysfunction.

Anemia was defined as a hemoglobin level < 11 g/dL and was further classified as mild (10.0–10.9 g/dL) or moderate (7.0–9.9 g/dL). Hemoglobin values used for anemia classification were obtained from routine laboratory investigations performed prior to delivery. Birth weight was categorized as low birth weight (<2500 g), normal birth weight (2500–3999 g), and macrosomia (≥4000 g). The mode of delivery was classified as vaginal or cesarean delivery.

OGTT results were available for all patients included in the study. Although not all patients received regular antenatal follow-up, gestational diabetes screening was performed during routine pregnancy assessments or other healthcare encounters occurring between 24 and 28 weeks of gestation. As no abnormal results were identified, gestational diabetes was not observed in the study population and was therefore not included in further analyses.

### 2.5. Statistical Analysis

All statistical analyses were performed using IBM SPSS Statistics (version 26.0, IBM Corp., Armonk, NY, USA). Descriptive statistics were used to summarize the characteristics of the study population, with categorical variables expressed as absolute frequencies and percentages.

Associations between categorical variables were assessed using contingency tables (crosstabulation). The Chi-square test (Pearson’s Chi-square) was used to evaluate statistical significance, while Fisher’s exact test was applied when expected cell counts were less than 5. The strength and direction of associations were further explored using measures of association such as the odds ratio (OR) with 95% confidence intervals (CI), as well as correlation coefficients (Pearson’s R and Spearman’s rho), where applicable. A *p*-value of <0.05 was considered statistically significant. For some variables, inferential statistical measures such as chi-square test, odds ratios, and confidence intervals could not be reliably calculated due to small subgroup sizes, the presence of zero or low-frequency cells, or the distribution of variables across multiple categories, which limited the validity of these analyses.

## 3. Results

**Figure 2:**
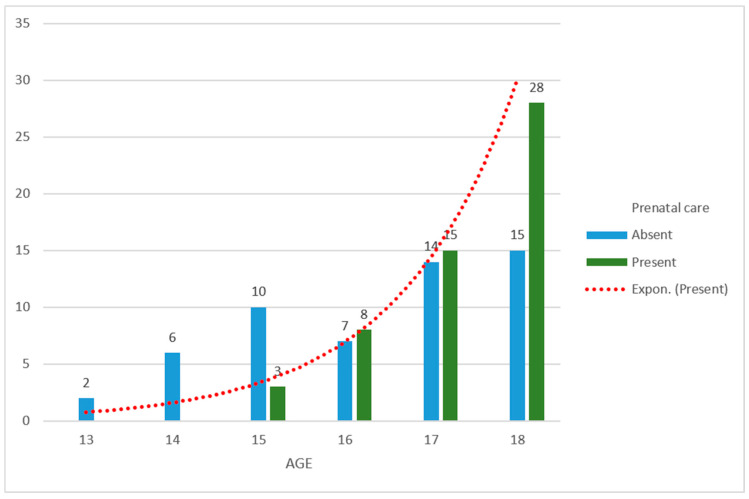
Prenatal care attendance by maternal age group.

General characteristics, including maternal age, mode of delivery, and preterm birth, were analyzed according to prenatal care status in order to assess baseline differences between patients with adequate prenatal care and those without adequate prenatal care (Table 3). Maternal age showed a statistically significant association with prenatal care status (*p* = 0.009), with younger adolescents being less likely to receive adequate antenatal follow-up. Patients aged 13–16 years were predominantly included in the group without adequate prenatal care (69.4%), whereas patients aged 17 and 18 years were more frequently included in the adequate prenatal care group (51.7% and 65.1%, respectively). (Figure 2).

Mode of delivery also differed significantly between the two groups (*p* = 0.001). Vaginal delivery was more common among patients with adequate prenatal care (66.7%), whereas cesarean section was more frequent among patients without adequate prenatal care (64.8%). Preterm birth was significantly associated with prenatal care status (*p* = 0.006; Fisher’s exact test *p* = 0.013), being more frequent among patients without adequate prenatal care (*n* = 14) compared with those with adequate prenatal care (*n* = 2). No significant association was identified between area of residence and prenatal care status (χ^2^ = 0.623, *p* = 0.430), with a relatively balanced distribution of patients with adequate prenatal care in both urban (53.0%) and rural settings (45.2%). Family educational level showed a strong association with prenatal care utilization (χ^2^ = 22.87, *p* < 0.001). Patients from families with higher educational attainment were more frequently included in the adequate prenatal care group (67.6%), whereas those from lower educational backgrounds were predominantly included in the group without adequate prenatal care (80.0%). A similar pattern was observed for maternal educational level, which showed a statistically significant association with prenatal care status (Fisher’s exact test, *p* = 0.003). All patients with primary education were included in the group without adequate prenatal care (100%), whereas all patients with adequate prenatal care had at least secondary education.

Maternal complications were analyzed according to prenatal care status. Maternal infections were significantly more frequent among patients without adequate prenatal care compared with those who received adequate prenatal follow-up (24.1% vs. 9.3%, *p* = 0.039). Hemorrhage was more frequently observed among patients with adequate prenatal care (Table 4).

Hypertensive disorders, including preeclampsia and gestational hypertension, were identified exclusively among patients without adequate prenatal care (7.4% each); however, no statistically significant association was identified (*p* = 0.118). Similarly, placental abruption was observed only among patients without adequate prenatal care, without reaching statistical significance (*p* = 0.495).

No significant differences were identified in the distribution of gestational edema between groups (14.8% vs. 13.0%, *p* = 0.781). Maternal anemia was highly prevalent in both groups, with comparable frequencies among patients with adequate prenatal care and those without adequate prenatal care (77.8% vs. 72.2%, *p* = 0.505). The severity of anemia was similarly distributed between the two groups, without a statistically significant association (χ^2^ = 0.67, *p* = 0.716).

Obstetrical complications were analyzed according to prenatal care status. Labor dystocia showed no significant differences between patients with adequate prenatal care and those without adequate prenatal care (14.8% vs. 9.3%, *p* = 0.375) (Table 5). Placenta previa was identified exclusively among patients without adequate prenatal care (9.3%), with no cases observed in the adequate prenatal care group, without reaching statistical significance (*p* = 0.057). Placental abruption was also observed only among patients without adequate prenatal care (3.7%), without a statistically significant association (*p* = 0.495). Cephalopelvic disproportion was significantly more frequent among patients without adequate prenatal care (*p* = 0.034). Hemorrhage was significantly associated with prenatal care status and was more frequently observed among patients with adequate prenatal care (59.3% vs. 35.2%, *p* = 0.012).

No significant differences were identified in the distribution of intrauterine growth restriction between groups (5.6% vs. 1.9%, *p* = 0.618). Neonatal respiratory distress was more frequent among patients without adequate prenatal care (35.2% vs. 20.4%, *p* = 0.086), while fetal distress also showed a higher frequency in this group (20.4% vs. 7.4%, *p* = 0.051), although no statistically significant associations were identified. Birth weight categories did not differ significantly between groups (*p* = 0.083), although low birth weight was more common among patients without adequate prenatal care (16.7% vs. 3.7%). No significant differences were observed in the distribution of neonatal jaundice between groups (46.3% vs. 42.6%, *p* = 0.699) (Table 6).

## 4. Discussion

The lower rate of prenatal care among younger adolescents may reflect increased vulnerability related to reduced health literacy, limited autonomy, social stigma, and broader socioeconomic constraints. The absence of antenatal follow-up may therefore reflect structural barriers rather than isolated patient decisions. Bucher et al. emphasized that maternal and perinatal outcomes result from multiple interacting factors and are not uniformly distributed across populations, highlighting the importance of analyzing individual complications separately [15].

The association between prenatal care and preterm birth represents one of the most consistent findings of the present study. The lower frequency of preterm deliveries among patients with adequate prenatal care may reflect a beneficial association with antenatal monitoring, in line with findings reported by Abanga et al. [7] This relationship should be interpreted cautiously, as prenatal care may also reflect underlying social stability and healthcare accessibility. The observed association with family educational level further supports this interpretation.

Maternal and obstetrical complications were not uniformly distributed between groups. Maternal infections were more frequent among patients without adequate prenatal care, possibly reflecting delayed diagnosis or inadequate management. Similar findings were reported by Gaikwad et al. [6], while Abanga et al. highlighted the importance of antenatal screening and timely management of maternal conditions [7]. 

The higher rate of cesarean delivery among patients without adequate prenatal care may reflect the increased frequency of emergency presentation, fetal distress, cephalopelvic disproportion, delayed obstetrical assessment, and limited opportunity for antenatal delivery planning. This pattern is consistent with findings from Agarwal et al. and Gaikwad et al., who associated unregistered status with increased obstetrical risk and emergency interventions [6,8]. Antenatal care may allow earlier identification of risk factors and facilitate planned delivery. Hypertensive disorders and placental complications were observed exclusively among patients without adequate prenatal care. Sokou et al. demonstrated the strong association between hypertensive disorders and adverse maternal and neonatal outcomes [16]. Similarly, Barbosa et al. [17] highlighted their clinical relevance in relation to fetal growth and birth weight, whereas Derbisbek et al. reported variable findings across populations [13]. The present results are consistent with this heterogeneity and should be interpreted cautiously, given the limited number of cases. Yasin et al. further demonstrated the association between maternal infections and adverse neonatal outcomes [18]. The lack of significant differences for other complications suggests that some outcomes may be influenced by factors independent of prenatal care, reinforcing the need for cautious interpretation.

The relationship between prenatal care and neonatal outcomes may partly reflect the higher frequency of prematurity observed among patients without adequate prenatal care. Higher rates of respiratory distress and low birth weight followed the pattern described by Agarwal et al., who associated prematurity with increased neonatal morbidity [8]. Socol et al. similarly linked maternal complications to adverse neonatal outcomes, particularly prematurity and low birth weight [19], while Barbosa et al. reported lower maternal and neonatal morbidity among patients receiving complete prenatal care [17]. Although neonatal complications were more frequent among patients without adequate prenatal care, the present study did not formally evaluate mediation, and these findings should therefore be interpreted as associations rather than causal mechanisms.

Hemorrhage showed a less consistent distribution. Although more frequently identified among patients with adequate prenatal care, this finding may have been influenced by differences in case mix, as high-risk pregnancies are more likely to receive specialized monitoring and documentation. Consequently, the observed association should be interpreted cautiously [17].

No association was identified between prenatal care and conditions such as gestational edema or anemia. Ramegowda described anemia as a common complication in adolescent pregnancies [20], which aligns with the high prevalence observed in this cohort. While Gaikwad et al. reported higher rates among unregistered patients [6], the present findings suggest that conditions such as anemia may be influenced by multiple nutritional and socioeconomic determinants beyond prenatal care alone.

The pattern of obstetrical complications suggests that the association between prenatal care and outcomes varies according to the underlying pathophysiological mechanisms. Labor dystocia showed no differences between groups, indicating a predominant influence of intrapartum factors. In contrast, placenta previa and placental abruption were observed only among patients without adequate prenatal care, although no statistically significant associations were identified due to the limited number of cases. Cephalopelvic disproportion was more frequent among patients without adequate prenatal care and represented the only obstetrical complication significantly associated with prenatal care status. This finding may reflect the importance of antenatal assessment in identifying maternal and fetal characteristics associated with delivery-related risks.

Pregnancy and neonatal outcomes showed a more consistent relationship with prenatal care, particularly regarding preterm birth. The higher frequency of prematurity among patients without adequate prenatal care suggests that lack of antenatal follow-up may be associated with adverse perinatal outcomes. Li et al. demonstrated an association between gestational age and neonatal complications, supporting the importance of prematurity in adverse neonatal evolution [21].

Neonatal complications, including respiratory distress, fetal distress, and low birth weight, were more frequent among patients without adequate prenatal care, although statistical significance was not reached. Agarwal et al. linked respiratory distress to prematurity [8], while Abanga et al. reported lower rates of low birth weight among patients receiving adequate prenatal care [7]. Derbisbek et al. further highlighted the contribution of nutritional and socioeconomic factors to adverse neonatal outcomes in adolescent pregnancies [13]. No association was identified between prenatal care and intrauterine growth restriction or neonatal jaundice, suggesting that these outcomes may be influenced by additional biological and diagnostic factors.

Although no association was observed between area of residence and prenatal care, educational background emerged as a more consistent determinant. Adolescents from families with lower educational attainment were less likely to receive prenatal care, a finding consistent with the observations of Socol et al., who associated lower educational and socioeconomic status with reduced prenatal care utilization and increased pregnancy complications [19]. These findings support the concept that prenatal care utilization may partly reflect broader social vulnerability.

The observed associations should also be interpreted in the context of potential residual confounding. Factors such as socioeconomic status, nutritional deficiencies, family support, and healthcare accessibility were not fully adjusted for and may have influenced both prenatal care utilization and pregnancy outcomes. Therefore, prenatal care status may partly reflect broader social vulnerability rather than an isolated clinical factor.

Recent evidence further supports the complexity of adolescent pregnancy outcomes and highlights the influence of additional biological, environmental, and socioeconomic factors. A study conducted in rural China demonstrated that elevated ambient temperature during pregnancy, particularly in the final weeks of gestation, was associated with an increased risk of preterm birth and reduced gestational age among adolescent mothers [22]. These findings emphasize the increased vulnerability of adolescent pregnancies and support the concept that adverse perinatal outcomes are multifactorial and may be influenced by both medical and environmental exposures.

The literature regarding pregnancy complications among adolescent mothers remains relatively limited compared with adult populations, particularly in relation to specific maternal and obstetrical complications. Most available studies focus predominantly on general neonatal outcomes such as prematurity or low birth weight, while fewer analyses evaluate the interaction between prenatal care, maternal complications, socioeconomic vulnerability, and obstetrical outcomes in adolescent primiparas. This limited evidence base further highlights the importance of studies investigating adolescent pregnancies, especially in socially vulnerable populations, where multiple interacting factors may influence both maternal and neonatal prognosis.

### Limitations

The retrospective design limits causal inference and depends on the accuracy and completeness of medical records.

The relatively small sample size reduced statistical power, particularly in subgroup analyses.

The study did not include multivariable adjustment for socioeconomic, nutritional, or healthcare-access factors, which may have acted as confounding variables.

Diagnostic variability may have influenced the identification of conditions dependent on antenatal monitoring.

## 5. Conclusions

The present study highlights the association between prenatal care and the distribution of maternal and obstetrical complications among primiparous adolescent patients. The higher frequency of complications such as maternal infections and cephalopelvic disproportion among patients without adequate prenatal care suggests that the absence of antenatal follow-up may be associated with delayed identification and management of maternal conditions. Hypertensive and placental disorders were observed exclusively among patients without adequate prenatal care, although no statistically significant association was identified. In this context, pregnancy and neonatal outcomes should be interpreted as potential consequences of maternal and obstetrical complications rather than independent endpoints. The differences identified between adolescents with adequate prenatal care and those without adequate prenatal follow-up underline the importance of early and continuous antenatal monitoring, particularly in a vulnerable population characterized by biological immaturity and social barriers to healthcare access. Improving access to antenatal services and addressing the socioeconomic determinants influencing healthcare utilization may represent important steps toward reducing complication rates and improving pregnancy outcomes among adolescent primiparas.

## Figures and Tables

**Table 1:** Baseline characteristics of the study population (*n* = 108).

Variable	Category		*n* (%)
Age (years)	Mean ± SD/Median/Mode	16.78 ± 1.33/17/18	108 (100%)
Age	13		2 (1.9%)
	14		6 (5.6%)
	15		13 (12%)
	16		15 (13.9%)
	17		29 (26.9%)
	18		43 (39.8%)
Residence	Rural		66 (61.1%)
	Urban		42 (38.9%)
Number of pregnancies	Primiparous		108 (100%)
Maternal education	Primary education (≤8 years)		9 (8.3%)
	Secondary education or higher		99 (91.7%)
Family educational level	Low educational level (≤8 years)		40 (37.0%)
	Higher educational level (≥high school)		68 (63.0%)
Smoking status	Smokers		0 (0%)
	Non-smokers		108 (100%)
Antenatal follow-up	YES		54 (50%)
	NO		54 (50%)
Gestational age at delivery	<37		16 (14.8%)
	37–42		92 (85.2%)
	>42		0 (0%)
Mode of delivery	Vaginal delivery		55 (50.9%)
	Cesarean section (C-section)		53 (49.1%)
Obesity	Present		3 (2.8%)
	Absent		105 (97.2%)

**Table 2:** Baseline characteristics of the study population.

Variable	Category	*n* (%)
Maternal Complications		
Gestational hypertension	Present	4 (3.7%)
	Absent	104 (96.3%)
Gestational edema	Present	15 (13.9%)
	Absent	93 (86.1%)
Preeclampsia	Present	4 (3.7%)
	Absent	104 (96.3%)
Hemoglobin	Normal hemoglobin: 11–13 g/dL	36 (33.3%)
	Mild anemia: 10–10.9 g/dL	38 (35.2%)
	Moderate anemia: 7–9.9 g/dL	34 (31.5%)
Hematocrit	Low hematocrit: <33%	43 (39.8%)
	Normal hematocrit: 33–44%	65 (60.2%)
Anemia	Present	81 (75%)
	Absent	27 (25%)
Maternal infections	Present	18 (16.7%)
	Absent	90 (83.3%)
Oral glucose tolerance test	NORMAL	108 (100%)
Obstetrical Complications		
Placenta previa	Present	5 (4.6%)
	Absent	103 (95.4%)
Placental abruption	Present	2 (1.9%)
	Absent	106 (98.1%)
Labor dystocia	Present	13 (12%)
	Absent	95 (88%)
Cephalopelvic disproportion	Present	23 (21.3%)
	Absent	85 (78.7%)
Hemorrhage	Present	51 (47.2%)
	Absent	57 (52.8%)
Pregnancy Outcomes		
Intrauterine growth restriction	Present	4 (3.7%)
	Absent	104 (96.3%)
Birth Weight	Low birth weight: <2500 g	11 (10.2%)
	Normal birth weight: 2500–3999 g	95 (88.%)
	Macrosomia: ≥4000 g	2 (1.9%)
Fetal distress	Present	15 (13.9)
	Absent	93 (86.1%)
Neonatal respiratory distress	Present	30 (27.8%)
	Absent	78 (72.2%)
Apgar score at 5 min	10	10 (9.3%)
	9	71 (65.7%)
	8	19 (17.6%)
	7	7 (6.5%)
	6	0 (0%)
	5	1 (0.9%)
Neonatal jaundice	Present	48 (44%)
	Absent	60 (55.6%)

**Table 3:** Comparison of general characteristics according to prenatal care status.

Variable	Antenatal Follow-Up	No Antenatal Follow-Up	χ^2^	*p*	OR (95% CI)
Maternal age group			15.8	0.009	
13–16 years	11 (30.6%)	25 (69.4%)			
17 years	15 (51.7%)	14 (48.3%)			
18 years	28 (65.1%)	15 (34.9%)			
Mode of delivery			10.7	0.001	0.27 (0.12–0.60)
Vaginal delivery	36 (66.7%)	19 (35.2%)			
Cesarean section	18 (33.3%)	35 (64.8%)			
Preterm birth			7.485	0.006	
Absent	52 (96.3%)	40 (74.1%)			
Present	2 (3.7%)	14 (25.9%)			
Residence			0.623	0.430	0.73 (0.33–1.59)
Urban	35 (53.0%)	31 (47.0%)			
Rural	19 (45.2%)	23 (54.8%)			
Maternal education			9.82	0.002	
Primary education (≤8 years)	0 (0%)	9 (100%)			
Secondary education or higher	54 (54.5%)	45 (45.5%)			
Family educational level			25.7	<0.001	8.36 (3.31–21.12)
Low educational level (≤8 years)	8 (20.0%)	32 (80.0%)			
Higher educational level (≥high school)	46 (67.6%)	22 (32.4%)			

**Table 4:** Association between prenatal care and maternal complications.

Variable	Antenatal Follow-Up	No Antenatal Follow-Up	χ^2^	*p*	OR (95% CI)
Gestational edema			0.077	0.781	1.17 (0.39–3.48)
Absent	46 (85.2%)	47 (87.0%)			
Present	8 (14.8%)	7 (13.0%)			
Maternal infections			4.27	0.039	0.32 (0.11–0.98)
Absent	49 (90.7%)	41 (75.9%)			
Present	5 (9.3%)	13 (24.1%)			
Gestational hypertension			4.1	0.118	-
Absent	54 (100%)	50 (92.6%)			
Present	0 (0%)	4 (7.4%)			
Preeclampsia			4.1	0.118	-
Absennt	54 (100%)	50 (92.6%)			
Present	0 (0%)	4 (7.4%)			
Anemia			0.44	0.505	0.74 (0.31–1.78)
Absent	12 (22.2%)	15 (27.8%)			
Present	42 (77.8%)	39 (72.2%)			

**Table 5:** Association between prenatal care and obstetrical complications.

Variable	Antenatal Follow-Up	No Antenatal Follow-Up	χ^2^	*p*	OR (95% CI)
Labor dystocia			0.79	0.375	1.70 (0.52–5.59)
Absent	46 (85.2%)	49 (90.7%)			
Present	8 (14.8%)	5 (9.3%)			
Hemorrhage			6.28	0.012	2.68 (1.23–5.84)
Absent	22 (40.7%)	35 (64.8%)			
Present	32 (59.3%)	19 (35.2%)			
Placenta previa			5.2	0.057	-
Absent	54 (100%)	49 (90.7%)			
Present	0 (0%)	5 (9.3%)			
Placental abruption			2.03	0.495	-
Absent	54 (100%)	52 (96.3%)			
Present	0 (0.0%)	2 (3.7%)			
Cephalopelvic disproportion			4.48	0.034	0.35 (0.13–0.95)
Absent	47 (87.0%)	38 (70.4%)			
Present	7 (13.0%)	16 (29.6%)			

**Table 6:** Association between prenatal care and pregnancy and neonatal outcomes.

Variable	Antenatal Follow-Up	No Antenatal Follow-Up	χ^2^	*p*	OR (95% CI)
Intrauterine growth restriction			1.03	0.308	-
Absent	51 (94.4%)	53 (98.1%)			
Present	3 (5.6%)	1 (1.9%)			
Fetal distress			3.79	0.051	-
Absent	50 (92.6%)	43 (79.6%)			
Present	4 (7.4%)	11 (20.4%)			
Neonatal respiratory distress			2.95	0.086	0.47 (0.20–1.12)
Absent	43 (79.6%)	35 (64.8%)			
Present	11 (20.4%)	19 (35.2%)			
Birth weight category			4.97	0.083	-
Normal	51 (94.4%)	44 (81.5%)			
Low birth weight	2 (3.7%)	9 (16.7%)			
Macrosomia	1 (1.9%)	1 (1.9%)			
Neonatal jaundice			0.15	0.699	1.16 (0.54–2.48)
Absent	31 (57.4%)	29 (53.7%)			
Present	23 (42.6%)	25 (46.3%)			

## Data Availability

The original contributions presented in this study are included in the article. Further inquiries can be directed to the corresponding authors.
